# Analysis of the causes of inferiority feelings based on social media data with Word2Vec

**DOI:** 10.1038/s41598-022-09075-2

**Published:** 2022-03-25

**Authors:** Yu Liu, Chen Xu, Xi Kuai, Hao Deng, Kaifeng Wang, Qinyao Luo

**Affiliations:** 1 Institute of Environment and Development, Guangdong Academy of Social Sciences, Guangzhou, 510635 China; 2 School of Geosciences and Info-Physics, Central South University, Changsha, 410083 China; 3 Research Institute for Smart Cities & Shenzhen Key Laboratory of Spatial Information Smart Sensing and Services, Shenzhen University, Shenzhen, 518060 China; 4 School of Optical Electrical and Computer Engineering, University of Shanghai for Science and Technology, Shanghai, 200082 China

**Keywords:** Human behaviour, Public health, Quality of life, Psychology and behaviour

## Abstract

Feelings of inferiority are complex emotions that usually indicate perceived weakness and helplessness. A lack of timely and effective interventions may *bring* serious consequences to individuals with inferiority feelings. Due to privacy concerns, those people often hesitate to seek face-to-face help, but they usually spontaneously share their feelings on social media, which makes social media a good resource for ample inferiority-related data. We randomly selected a sample of posts indicating inferiority feelings to explore the causes of inferiority. Through language analysis and natural language processing, we constructed a Word2Vec model of inferiority based on social media data and applied it to the cause analysis of inferiority feelings. The main causes of inferiority feelings are personal experience, social interaction, love relationship, etc. People feeling inferior about their personal experiences usually are largely influenced by their ways of thinking and life attitudes. Social and emotional factors overlap somewhat in the development of inferiority. In love relationships, males are more prone to inferiority feeling than females. These findings will help relevant institutions and organizations better understand people with inferiority feelings and facilitate the development of targeted treatment for those with potential self-esteem problems.

## Introduction

Inferiority feelings are complex emotions that usually indicate perceived weakness and helplessness^1,2^. People with low self-esteem often feel depressed. Severe inferiority feelings can easily lead to various diseases, such as endocrine disorders, decreased immunity, and even depression^3,4^. For most individuals, receiving professional treatment does help. To receive targeted treatment, first, these individuals must communicate to relevant medical institutions the true causes of their inferiority feelings. However, due to privacy concerns, these individuals are often reluctant to seek face-to-face help, resulting in not only delay in treatment but also difficulties for scholars to understand and study inferiority^5,6^. Therefore, it is necessary to adopt a passive, targeted approach to determine the causes of individuals’ inferiority feelings.

Fortunately, the rise of social media in recent years has provided a new way for us to investigate the target population in a passive and targeted state and to understand and study the causes of inferiority feelings. On one hand, the openness of social media makes it a common tool for users to express their emotions and communicate with each other. On the other hand, the anonymity of social media makes users feel free and safe to talk about private issues, such as physical illnesses and mental disorders^7,8^. As one of the largest and most popular social media in China, Sina Weibo has more than 500 million users, 80% of which are young people (10–40 years old), and more than half of the total users have bachelor’s degrees or above^9^. More importantly, Weibo has more than 100 million daily posts, covering topics ranging from news, weather, emotions, and medical treatment to comments on social activities^8,10^. The diversity of Weibo content makes it an excellent platform for companies to build brands and the government to be engaged in people’s livelihood; the openness of topics makes Weibo an effective tool for people to express their emotions and communicate without being face-to-face, and the anonymity and the confidentiality of user information makes Weibo an important space for many patients with diseases to exchange their experiences^11,12^. Earlier, efforts have been made by some scholars to use Weibo data in AIDS-related research. Recent studies have also indicated that social media can be used to study psychological problems, such as anxiety and depression^13,14^. Recently, it has been discovered that there is a large quantity of data, rich in semantic information, published by individuals with inferiority feelings on social media. These data have laid a foundation for scholars to study the characteristics of the inner activities and the causes of inferiority feelings.

However, existing research has mostly used manual interpretation to process data and then establishes a series of coding schemes, offering a practical way to study psychological problems via social media^14,15^. Although manual coding can be used to classify social media data macroscopically, given the large amount of data, it is difficult to fully consider the semantic information hidden behind the data using this method^13,16^. Moreover, for a specific research topic, some relevant knowledge can be provided by the semantic primitives or vocabularies used to express the topic; however, the most pertinent knowledge is often implicit in the semantics of those primitives or vocabularies^14,16^. Hence, a professional background is essential for obtaining subject knowledge that truly reflects a specific topic^7,17^. Therefore, it is necessary to establish a semantic model of background knowledge on a specific topic to obtain semantic primitives or vocabularies that effectively represent the domain. In this study, we perform a subject classification of inferiority feelings using artificial coding. According to the characteristics of the relevant social media data, natural language processing technology is applied to construct a self-esteem semantic emotion model based on social media data to extract and visualize the highly represented semantic primitives. Next, we analyze the causes of inferiority feelings at a fine-grained semantic level. Our research results will help relevant institutions and organizations better understand people with inferiority feelings in an effort to develop targeted treatment for those with potential self-esteem problems.

## Data

Public datasets were used in this study. Specifically, when people register an account on Sina Weibo (also called Weibo), they are informed that they can privatize or publicize their posts as they wish, and only public posts can be viewed and downloaded over the platform. Therefore, in this research, we used only public Weibo posts as a data source. The data acquisition process consisted of two steps: data collection and data preprocessing.

### Data collection

In this study, the Scrapy1.5.0 application framework was used to customize a keyword-based crawler program to collect Weibo data. Scrapy, written in Python, is a mainstream web crawler development framework. It provides a series of basic classes of crawlers, on which users can extend their custom functions. The specific workflow is as follows.

First, we registered a Sina Weibo account and logged in, simulated a user inputting keywords into the search box of Weibo, and then precisely analyzed the returned website source code. Second, to accurately crawl the Weibo posts from January 1, 2011, to January 1, 2018, we used RequestURL to obtain the HTML of the login page and the lxml4.2.1 Library in Python to analyze the code, and we gathered the data of user attributes such as username, followers, follows and their number, content, and dates of posts. An explanation of the selection of keywords is necessary. There are two types of inferiority-related posts on Sina Weibo: posts containing the keyword “inferiority” and posts without the keyword “inferiority” but that reveal an intense perception of inferiority. Thus, the results returned from a search based only on the keyword “inferiority” were not comprehensive. To ensure comprehensiveness, two different data crawling strategies were adopted. The specific steps are as follows.

For the first types of inferiority-related posts, we searched directly with the keyword “inferiority”. With regard to the second type of posts, a formal ontological analysis was used to search for them. Ontologies are clear and comprehensive statements on a specific concept that convey the perception of the nature of objects or concepts. By disassembling and interpreting the nature of concepts, formal ontological analysis can realize sharing and reconstruction of multisource heterogeneous information and engender perceiving the nature of multisource information (for a description of a formal analysis method based on ontology, please refer to references Tan et al. and Kuai et al.^18,19^). This approach has been widely applied for word meaning disambiguation, concept matching, and information architecture and has socioeconomic benefits^20,21^. Specifically, we identified the inferiority feelings to be “despising oneself”, “feeling inferior to others” and being “down in spirits” which combined the definitions of inferiority feelings from Rendian, Cihai, Modern Han Language Word Dictionary, and Baidu Encyclopedia. Ontological attributes of inferiority feelings could be extracted from the definition through semantic analysis based on the language technology platform (LTP) developed by the Harbin Institute of Technology; these attributes are “feeling inferior to others,” feeling “depressed” and feeling “sad”. Additionally, to thoroughly search for the second type of post, we added synonyms of the attributes to the set of search keywords (Table 1), gathered the crawl data containing those keywords, and finally filtered the data according to Eq. (1):

1 $${\text{A}}_{{1}} \cap {\text{A}}_{{2}} \cap {\text{A}}_{{3}} \cap {\text{A}}_{{4}} \cap \, ({\text{A}}_{{5}} \cup {\text{A}}_{{6}} ).$$

**Table 1:** Attributes of inferiority feelings.

Keyword	Synonym	Group
认为 (think)	以为 (feel)	A1
自己 (oneself)	本人 (myself),我 (I)	A2
不如 (inferior to)	比不过 (not equal to)	A3
别人 (other people)	他 (he),她 (she),他/她们 (them)	A4
低落 (feel low)	沮丧 (depressed),颓唐 (dejected),萎靡 (downhearted),消沉 (despondent)	A5
悲伤 (sadness)	心酸 (feel sad),悲戚 (grief),伤感 (unhappiness),悲哀 (sadness),哀痛 (mourning)	A6

A_1_, A_2_, A_3_, A_4_, A_5_, and A_6_ are shown under “Group” in Table 1.

### Data preprocessing

The collected data contained noise, such as advertisements, inspirational stories, running comments, and discussion on specific topics. Therefore, the data required cleaning. The specific process is as follows.

First, according to previous research on Weibo, accounts with more than 5000 followers are generally marketing accounts, and their posts are mostly advertisements^22^. Therefore, we deleted posts by such accounts. A total of 2.40 million posts remained.

Second, not all downloaded posts were related to the research subject but were posts on soul soothers, philosophical thinking, practical comments, etc. A binary classifier was required to identify posts related to inferiority feelings. A supervised classification approach was adopted that required a training dataset. However, to the best of our knowledge, no public dataset is available^7,14^. Therefore, 20,000 posts were randomly selected from 2.40 million posts to mark the training data. To ensure faithful labeling, we recruited five participants to our research team with backgrounds in psychology to tag the posts. Specifically, four of them were evenly divided into two groups to label the posts independently, and all labeling results came from negotiations within the group. If no agreement was reached, the fifth member participated in the discussion, and the principle of the minority following the majority was observed. The results showed that 15,921 posts were related to inferiority (target posts), while the others were not (off-targeted posts). One thousand posts were extracted from each of the two types of posts as the validation dataset. Based on existing research results, the support vector machine (SVM) has been shown to achieve high accuracy in classification of Weibo^13,14^. Thus, an SVM was employed to distinguish the target posts. As a result, the posts were classified into the two categories of targeted and off-targeted. The posts were then vectorized as the input of the SVM and used to train the SVM model. Finally, the trained SVM model was used for data cleaning, with an accuracy of 80.38%.

Finally, we established the Weibo post pool (WPP) to save the nearly 1.2 million posts remaining for the research.

## Methodology

There are two parts in this section. First, “Themes” identifies the themes of the posts with the most frequent occurrence. Second, “Semantic characteristics” conducts semantic analysis on the cause of inferiority feelings based on the “Themes”.

It is sure that all the methods are conducted according to the relevant guidelines and regulations in this study.

### Themes

The 1.2 million posts from 2011 to 2017 in the WPP were classified into seven blocks by year. For each year we extracted 200 posts (for a total of 1400 posts) revealing the causes of inferiority feelings. To be clear, some posts in the WPP did not reveal the reasons for inferiority feelings. Therefore, we had three members in our team with ample experience in psychological research. Two of them sampled posts and discussed the whether to include the sample. When they had disagreement, they returned the post to the WPP and continued sampling until we had 200 samples for each year (for a total of 1400 samples). The two members discussed each post extracted, confirmed the most frequent themes, and created theme codebook^7,23^. The posts in the codebook were coded as follows: ① inferiority feelings about physical defects(IF-PD); ② inferiority feelings about love and affection(IF-LA); ③ inferiority feelings about family background(IF-FB); ④ inferiority feelings about personality(IF-P); ⑤ inferiority feelings about personal experiences(IF-PE); ⑥ inferiority feelings about social interaction(IF-SI); ⑦ inferiority feelings about learning(IF-L); ⑧ inferiority feelings about abilities(IF-A).

Then, two members categorized posts according to the codebook, during which any divergence was resolved through consensus. Finally, to verify the consistency of the compilation, the third research member randomly selected 140 posts to classify and assess the consistency of the codebook classifications using the Cohen's kappa coefficient. It is worth explaining that Cohen's kappa is a common index to measure classification accuracy and consistency test. Generally, its value is between 0 and 1. The closer it approaches 1, the higher the classification accuracy or consistency will be^25–26^. After the codebook category and consistency verification, the causes of inferiority feelings were identified as follows: ① IF-PD (kappa: 0.8027); ② IF-LA (kappa: 0.6835); ③ IF-FB (kappa: 0.6509); ④ IF-P (kappa: 0.7105); ⑤ IF-PE (kappa: 0.7731); ⑥ IF-SI (kappa: 0.7012); ⑦ IF-L (kappa: 0.8916); and ⑧ IF-A (kappa: 0.7531).

### Semantic characteristics

In “Themes”, we identified the themes of posts that revealed reasons for inferiority feelings through manual coding at a macro level. However, the posts contained copious semantic information. Previous studies have shown that for a concrete subject, real knowledge about the subject is implicit in the semantic primitives used to express it or in the information alluded to by vocabularies^19,27^. Therefore, a professional background in a certain field is indispensable to obtaining the real knowledge and semantic primitives of the subject. Thus, it is necessary to establish a semantic model of background knowledge of the subject and employ a semantic primitive extraction algorithm based on semantic association to obtain highly representative semantic primitives or vocabularies. Such an approach facilitates the exploration of the reasons for inferiority feelings at the micro semantic level.

**Figure 1:**
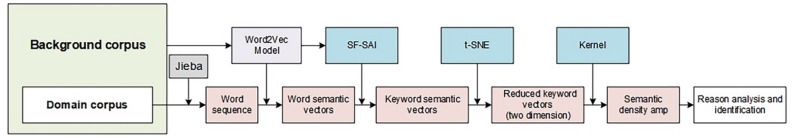
Flowchart of the semantic analysis of the causes of inferiority feelings (Drawing with WPS Office 2019^30^).

This research builds on the corpus of background knowledge consisting of the 1.2 million downloaded posts about inferiority described in “Data collection” and the corpus of field knowledge consisting of the posts labeled with the reasons for inferiority described in “Themes”. For the background knowledge corpus, we build a semantic model of inferiority by employing Word2Vec (Considering the data characteristics of this study, calculation efficiency, and accuracy of similarity modeling, we chose the skip-gram model for Word2Vec training. And according to the existing research, the search window was set to 10, the word vector dimension was set to 400)^16,28,29^. For the field knowledge corpus, we disassembled each post into sentence sequences and divided each sentence into word sequences by using the Jieba word segmentation tool, finally labeled the parts of speech of the words. In this way, the Word2Vec model mapped the word sequences to the high-dimensional word vectors, and we extracted the semantic primitives of the field knowledge corpus by using the Semantic Frequency-Semantic Active Index (SF-SAI) semantic primitive extraction algorithm based on the coherence of the text^16^. However, the extracted semantic primitive is a high-dimensional vector, and dimension reduction is indispensable for intuitive presentation. Thus, we employed the t-distributed stochastic neighbor embedding (t-SNE) algorithm to reduce high-dimensional to two-dimensional vectors. Finally, we performed a kernel core density analysis and drew a semantic atlas for the analysis of factors related to inferiority feelings. The specific steps are shown in Fig. 1. Among them, the *SF-SAI* and word dimensionality reduction visualization were shown in Appendix 1 and Appendix 2, respectively.

## Results and discussion

### Themes

Two members of the research team with ample psychology research experience randomly extracted 1400 posts that revealed causes of inferiority feelings from the WPP for each publication year (200 posts each year). Subsequently, subject analysis on the reasons for inferiority feelings derived from these posts was carried out, and the results are shown in Table 2. As shown, in 270 posts (19.29%), inferiority feelings were caused by personal experiences, which was the most common cause, while in 25 posts (1.79%), inferiority feelings were caused by family, which was the least common cause. Among the eight main causes of inferiority feelings, we selected the four causes with the highest proportion for further analysis.

**Table 2:** Causes of inferiority feelings.

Cause	Rate (%)	Examples
Inferiority feelings about learning	40 (2.86)	I feel too inferior to look at my English teacher in every class
Inferiority feelings about social interaction	110 (7.86)	Every time I’m in a crowded place, I feel so scared and inferior that I dare not to speak to others
Inferiority feelings about love and affection	247 (17.64)	I feel inferior to my brilliant girlfriend
Inferiority feelings about family background	25 (1.79)	My family environment has made me feel inferior since childhood
Inferiority feelings about physical defects	261 (18.64)	I haven’t been praised for my appearance, which makes me feel inferior
Inferiority feelings about abilities	238 (17)	Sometimes I feel shy, useless and inferior to others, which makes me feel more and more inferior
Inferiority feelings about personality	209 (14.93)	Being sensitive makes me lack security and feel inferior
Inferiority feelings about personal experiences	270 (19.29)	It is my childhood that makes me inferior; I took care of myself like a wild child without parents

### Inferiority feelings about personal experiences

**Figure 2:**
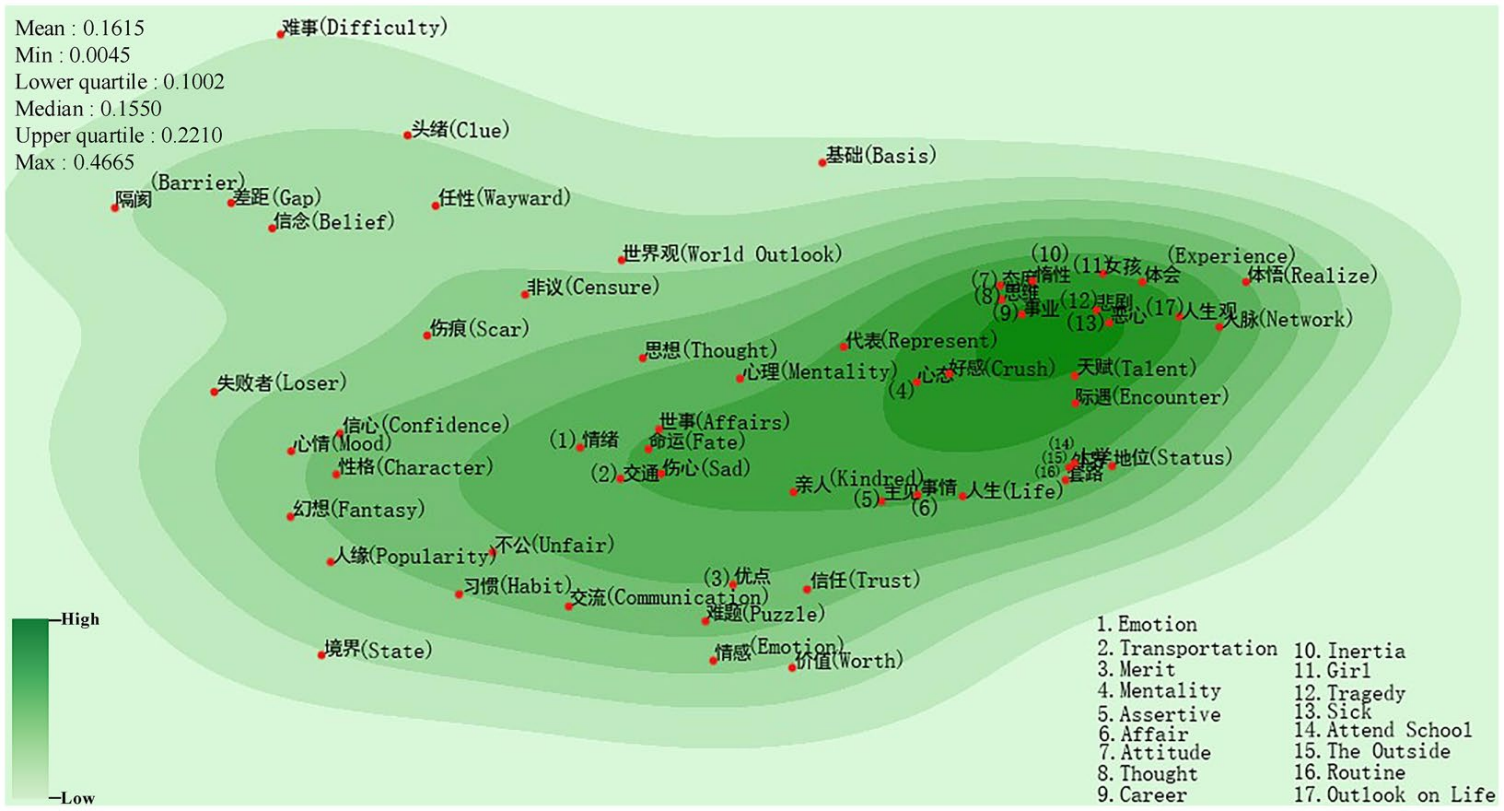
Semantic map of inferiority feelings about personal experience (Drawing with Seaborn 0.8.1 in python^36,37^).

Figure 2 is the semantic space of inferiority feelings caused by personal experience presented as a semantic density map. Semantic space is the space of linguistic meaning, which is a set of semantically related words. More specifically, semantic space is a set including domain concepts and their relationships^31^. In the semantic space, each concept or word is described as a point, and the semantic difference between two concepts is represented by distance, which is inversely proportional to the semantic relevance between two concepts^19,31^. The points represent the semantic primitives extracted with the *SF-SAI* algorithm. The minimum, maximum, quartile and median of the distance of the semantic primitives are in the upper left corner of the picture. Also, the semantic primitives are not evenly distributed in the semantic space, and the regions with high semantic density are darker, implying that the causes represented by the semantic primitives located in these regions are the main causes of inferiority. The distance of semantic primitives (usually called semantic distance) is a relative distance. The closer the distance between semantic primitives is, the higher their semantic relevance is^16^. Generally, two semantic primitives are considered semantically close if both the mean and the lower quartile of the distances among all semantic primitives in the same figure are bigger than the value of their distance^32,33^.

As shown in Fig. 2, the mean and the lower quartile are 0.1615, 0.1002, respectively. The central area is dotted with various semantic primitives including “事业 (Career)”, “态度 (Attitude)”, “思维 (Thought)”, “惰性 (Inertia)”, “悲剧 (Tragedy)” and “恶心 (Sick).” “Tragedy” is close to “Sick” (relative distance: 0.0091) and the mean value of the relative distance among “Thought”, “Attitude” and “Inertia” is 0.0109. According to the posts from which those primitives are extracted, many people’s frustrations in study, career, and life are due to their mindset problems or incorrect attitudes, which further leads to inferiority. We also see three closely located primitives of “外界 (The Outside)”,“地位 (Status)” and “套路 (Routine)” (mean: 0.0120) which are all related to social interaction. Usually, the corresponding posters care a great deal about their social status and others’ perceptions of them, so they often just show their strong aspects in front of others, which increases the probability of their failure and then leads to inferiority^34,35^. In addition, the closeness between “心态 (Mentality)” and “好感 (Crush)” (0.0096) indicates that some people manage to overcome negative emotions and develop a positive state of mind by self-adjusting or seeking the help of psychologists.

### Inferiority feelings about physical defects

**Figure 3:**
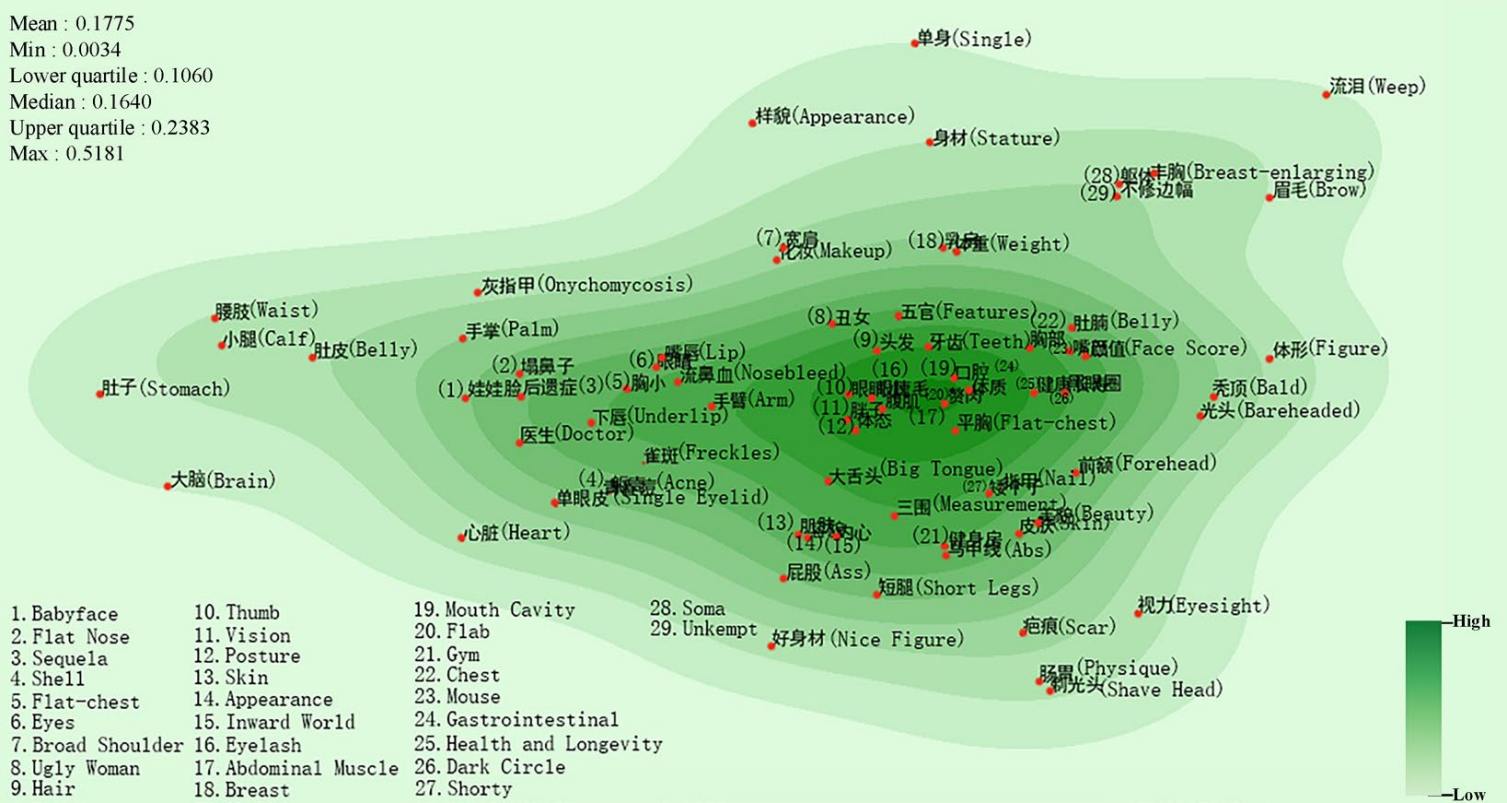
Semantic map of inferiority feelings about physical defects (Drawing with Seaborn 0.8.1 in python^36,37^).

Figure 3 shows the semantic map of inferiority feelings caused by physical factors and the mean value is 0.1775, while the lower quartile is 0.1060. As seen in the figure, “赘肉 (Flab)” and “腹肌 (Abdominal Muscle)” are positioned at the center of the map; the semantic primitives “abdominal muscles”, “胖子 (Fatty)” and “体态 (Posture)” are close to each other (mean distance: 0.0130). This indicates that people are most sensitive about their weight. Meanwhile, there are many semantic primitives in Fig. 3 that describe facial body parts, such as the teeth, mouth, eyelashes, and lips. These suggest that flaws in the parts of one’s face also cause inferiority feelings^38,39^. In contrast to previous general perception, the present study shows that the semantic primitives associated with height do not appear in semantically dense areas, demonstrating that height is not the main reason for perceived physical inferiority. Recent studies have shown that women with an appropriate amount of fat around the waist had lower chances of fractures^40,41^. This implies that some seemingly physical defects (such as "Flab ") that lead to people's inferiority are beneficial to their health.

Existing research shows that physical deficiencies usually affect individual's personality. In semantic map of inferiority feelings about personality(Appendix 3), “亲和力 (Affinity)”, “负面 (Contact)”, “情绪 (Emotion)”, “脾气 (Temper)”, “不合群 (Asocial)”, “怨念 (Resentment)” and “小气 (Pettiness)” are at the center of the semantic map. And “Affinity”, “Contact”, and “Emotion” are close to each other (mean distance: 0.0131). This indicates that people with inferiority for personality may be sensitive and inaccessible, and have negative moods, etc., which is also an important characteristic of people with inferiority for physical defects. Such people are very sensitive to others’ reactions toward themselves during social interactions. A wink, a few words, or an action by others can cause their emotional fluctuations. These emotional fluctuations tend to last for a long time, during which several negative emotions may occur^42,43^. Moreover, the semantic distances between “性格 (Character)”, “思维 (Thought)”, “羁绊 (Fetter)” and “悲观主义 (Pessimism)” are rather close, indicating that the inferiority feelings of such people are, to a great extent, influenced by their modes of thinking^44,45^.

### Inferiority feelings about love and affection

**Figure 4:**
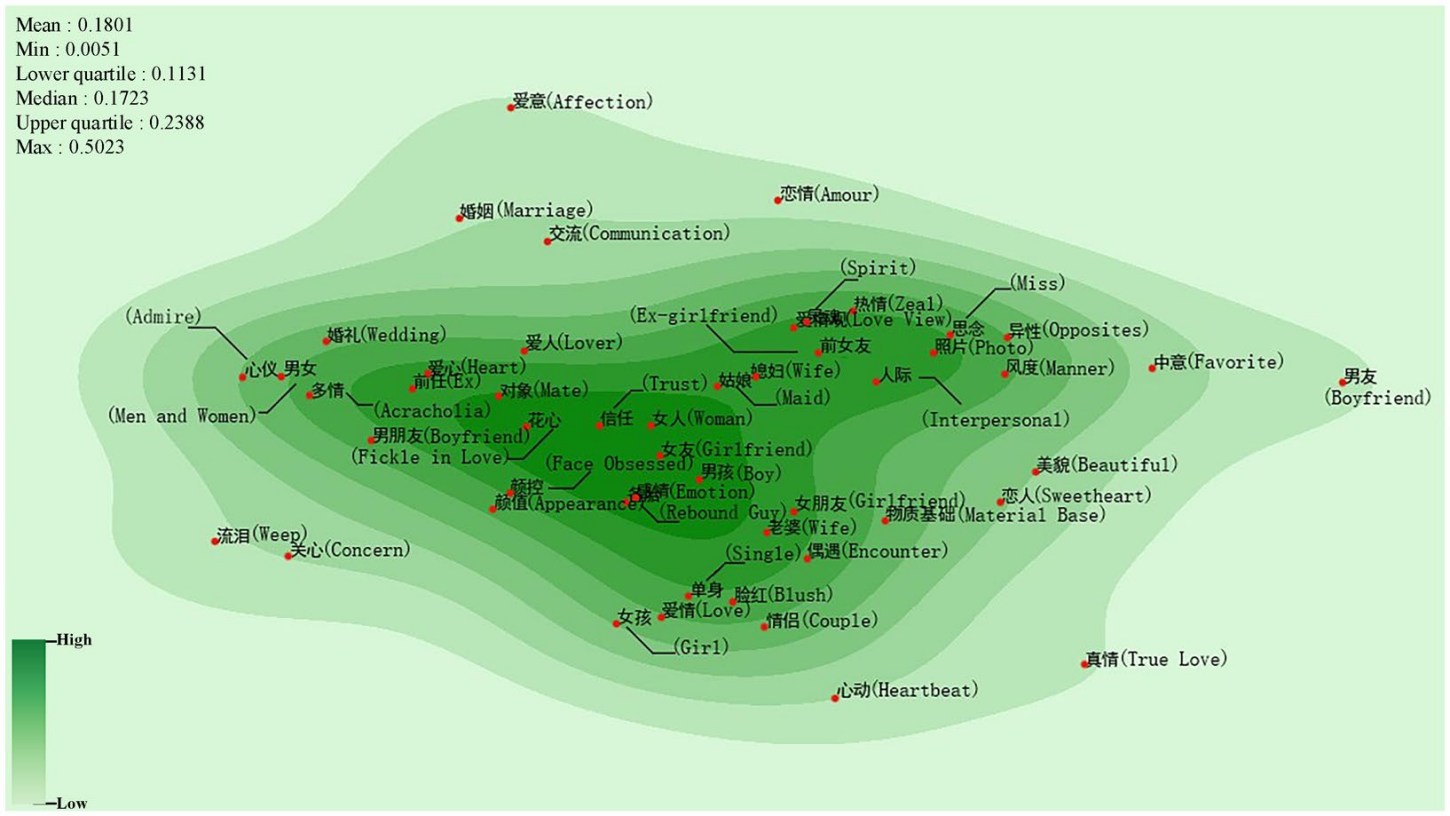
Semantic map of inferiority feelings about love and affection (Drawing with Seaborn 0.8.1 in python^36,37^).

Figure 4 shows the semantic map of inferiority feelings caused by affection and love. “信任 (Trust)”,“花心 (Fickle in Love)”,“感情 (Emotion)”, “备胎 (Rebound Guy)” and other semantic primitives about relationships are in semantically dense areas of the map. Inferiority feelings can undermine love and affection. Regarding affection and love issues, some people are devoid of the courage to express their inner feelings and dare not accept another’s love (posts such as “I am susceptible to feeling low esteem when I meet someone I like, and I dare not talk to him/her” are commonly found). Under such circumstances, psychological problems, such as anxiety, arise. Furthermore, some people lack a sense of security in their relationships; they do not trust their partners, and they always feel that they play the role of “Rebound Guy”. This phenomenon is reflected in posts such as “We have been together for more than 2 years, yet I always feel that I am just a Plan B. I truly feel inferior in the relationship.” These feelings of insecurity and distrust can lead to inferiority feelings in the relationship^46^. Moreover, notably, “女朋友 (Girlfriend)” and “男朋友 (Boyfriend)” refer to the person that the individual loves. However, the semantic primitive “Girlfriend” is positioned at the center of the semantic map, while “Boyfriend” is in a sparse area of the semantic map. This implies that men are more prone than women to harbor inferiority feelings in mutual relationships. Some research indicates that when one cannot fulfill the economic and social pressures of love relationships, men (or those playing the role of men) are more likely than women to feel inferior^44,47^. In addition, the semantic primitives related to appearance, such as “颜值 (Appearance)” and “Beauty,” are not positioned in the semantically dense areas. This indicates that appearance is not the most predominant factor giving rise to inferiority feelings about affection between men and women^48,49^. In summary, the anxieties caused by inherent confusion and emotional needs carry greater weight than their concern about appearance for people who are in love.

### Inferiority feelings about abilities

**Figure 5:**
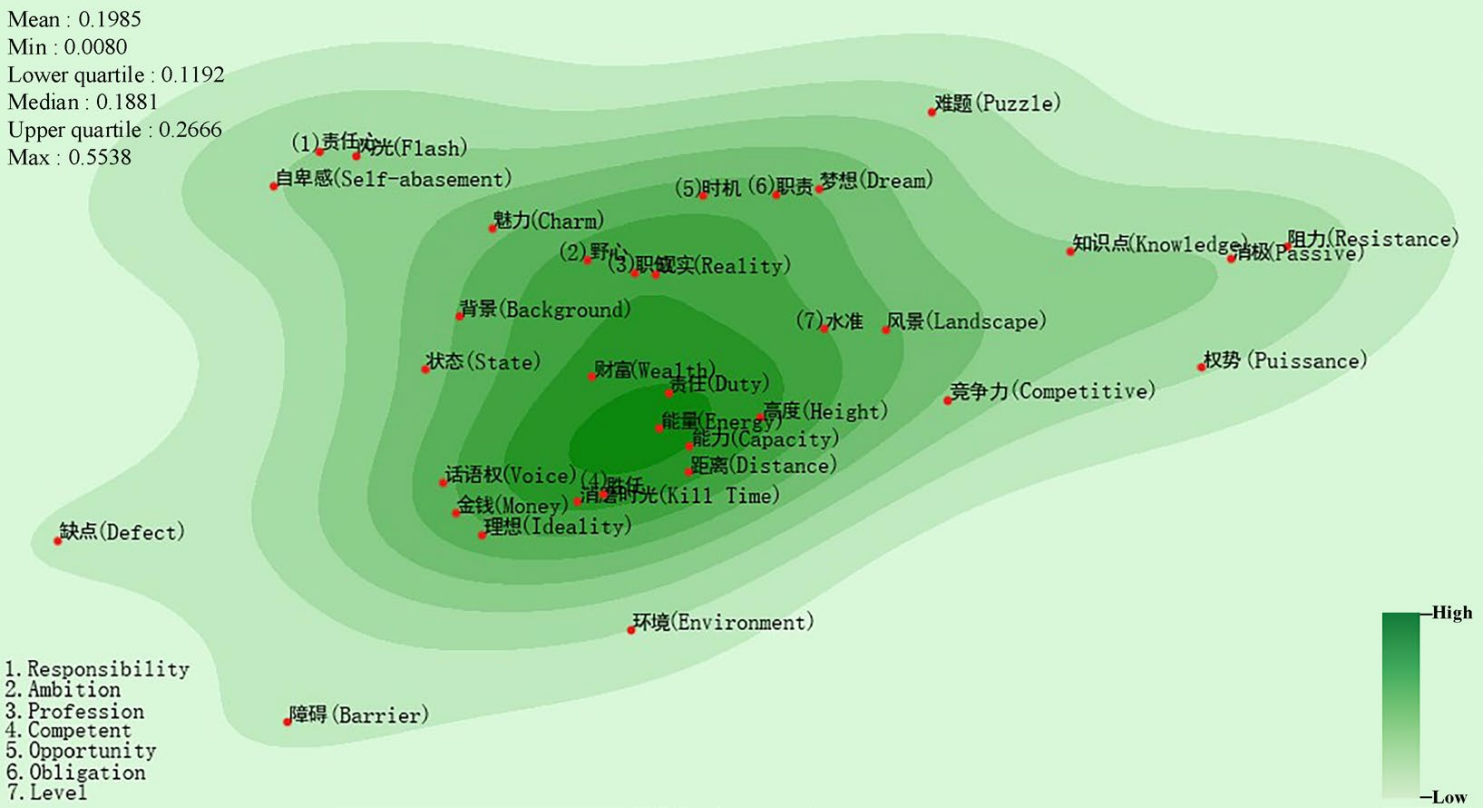
Semantic map of inferiority feelings about ability (Drawing with Seaborn 0.8.1 in python^36,37^).

In the semantic map of inferiority feelings about ability (Fig. 5), “责任 (Responsibility)”, “能量 (Power)” and “能力 (Capacity)” are distributed in the semantically dense area and close to one another (mean distance: 0.0306). The “pressure-state-response” model (PSR) from the field of ecological risk assessment can be used to describe people’s status in their social lives. Responsibility can be interpreted as social obligations, such as family responsibilities and work responsibilities, that individuals must assume. Capacity or power represents the various and necessary skills required of an individual to cope with pressure and to take responsibility in his or her social life. Working ability, learning ability, and social interaction ability are among these skills. These three semantic primitives describe the three aspects of an individual’s comprehensive abilities. People feel inferior when they are less competent in one or more of these three aspects. Generally, people of inferior ability are less able to adapt to the environment and to deal with frustration at work. Therefore, they tend to suffer from greater psychological pressure than others in the face of setbacks, such as unemployment, divorce, and illness^50,51^. Furthermore, in the event of unfair treatment, they are more sensitive and presume that others hold them in contempt, and easily feel offended.

In addition, as an important ability, social adaptability plays an important role in causing individual inferiority feelings. According to semantic map of inferiority feelings about social interaction as shown in Appendix 4,“交流 (Communication)” is at the center of the semantic map; “挫折 (Setback)” and “流眼泪 (Weep)” are the two closest semantic primitives on the semantic map (relative distance: 0.0038). This indicates that weeping is an important approach for people with inferiority feelings about social interaction to vent negative emotions when they suffer from setbacks. Such people are normally characterized as being emotionally fragile and introverted and having ineffectual communication skills. Moreover, the distribution of semantic primitives presents a “dual-core” formality in Appendix 4. The three primitives “泪水 (Tear)”, “内向 (Diffidence)” and “男朋友 (Boyfriend)” constitute a semantic subarea. Other primitives in their proximity include “女生 (Girl)” and “男生 (Boy)”. This shows that in social relationships, some inferiority feelings are caused by love and affection between boys and girls. Therefore, social and emotional factors have, to a certain extent, an overlapping effect on the development of inferiority feelings^49,52^.

## Conclusion

Based on social media data about inferiority, this study explores the causes of inferiority feelings. The results of the theme analysis show that inferiority feelings mainly stem from experience, physical defect, personality, love relationship, ability, social interaction and so on.

For people feeling inferior due to personal experience, their ways of thinking and life attitudes are the main internal causes of their inferiority feelings. Some of them tend to negatively evaluate themselves because of their failures and setbacks in life or work. Some of them hold their own distinctive views of themselves in the uncertain world and always pursue ultimate perfection in their work. Those self-evaluations and views will lead to their self-denials. Body inferiority are not primarily about height, some other physical defects were once thought to cause inferiority feelings, such as flabbiness. Actually, those so-called defects often serve as incentives for people to get better health. Individuals with physical deficiencies usually have characteristics like sensitivity, inaccessibility, and negative emotions. Such people should be encouraged and guided to learn to view problems from a developmental, long-term, positive perspective to eliminate their excessive pessimistic ideas and conceptions^44,45^. Compared with females, males are more prone to inferiority in love relationships, and, according to a survey, financial and societal pressures are the primary causes instead of appearance^44,53^. Ability and inferiority constitute an entia of contradictions. Incompetent people are usually characterized with self-esteem, and people with inferiority feelings often have a sense of inability. Additionally, people whose inferiority feelings come from social skills are usually weak in managing interpersonal relationships, the weakness makes them fear social situation and avoid heterosexual contact^49,52,54^. Considering that, relevant institutions and governments should implement necessary steps to guide them to a correct their self-images and thus help them out of the dilemma in which they feel trapped.

Despite the findings above, this study still has a few limitations worthy of our summarization and reflection. Firstly, the data in this study were obtained from Sina Weibo, so the conclusions may only be applicable to China. In addition, inferior individuals who do not use social media are not discussed. Secondly, since large-scale offline investigation of posters cannot be carried out, this study only reveals the strong correlation between inferiority feelings and social factors as well as emotional ones, the causal relationship has not been fully demonstrated, and the interrelationships between the causes are not fully revealed. Thirdly, due to limitations in methods and techniques, this study does not analyze posts failing to reveal inferiority causes.

Nevertheless, the method proposed in our study can provide professional institutions specializing in psychology with a relatively low-cost way to explore causes of inferiority feelings. Moreover, these findings will help relevant institutions and organizations better understand people with self-esteem and other mental health problems, thus facilitate the formulation or adoption of better targeted treatment.

## Supplementary Information

Supplementary Information.
