# Supplementary material for: Analysis of the causes of inferiority feelings based on social media data with Word2Vec
Source: Sci Rep. 2022 Mar 25;12:5218. doi: 10.1038/s41598-022-09075-2 (PMC8956725; doi:10.1038/s41598-022-09075-2)
Supplement: Supplementary file 1 — Supplementary Information. [file 41598_2022_9075_MOESM1_ESM.docx]

## Appendix 1: SF-SAI

Generally, the importance of a specific semantic primitive in a domain text is directly proportional to the number of times it appears in the domain text. The TF-IDF semantic primitives extraction algorithm, which is widely used at present, is based on this principle. Its idea is that the higher the frequency (TF) of a word in a text, and the lower the frequency (IDF) of a word in other text, the more important the semantic primitives are to the text. However, when the amount of text is very large, the recognition of semantic primitives extracted by the term frequency-inverse document frequency (TF-IDF) method is poor ^60^. In view of this situation, the term frequency-keyword active index (*TF-KAI*) algorithm has been proposed, which improves the *IDF* operation part of *TF-IDF* and improves the text identification. However, neither TF-IDF nor *TF-KAI* fully considers the semantic information and semantic association behind the semantic primitives. Recent studies have found that although semantic primitives with high frequency have generality, they lack domain representation in a specific field ^17^. Therefore, we must consider the semantic information and semantic association hidden behind the semantic primitives if we want to extract the semantic primitives representing domain knowledge. Based on this idea, researchers have proposed to extend TF to semantic frequency (SF) and KAI to the semantic active index (SAI) and have developed the SF-SAI algorithm^17,28^.

Compared to algorithms based on word frequency, *SF-SAI* can more efficiently extract the most representative semantic primitive in a certain field. For this reason, the *SF-SAI* algorithm was used in this research to extract the semantic primitives, exposing the reasons for inferiority. The *SF-SAI* algorithm can be expressed as

$SF-SAI=n(i_{C\_corpus},j)^{2}\times\frac{n(all)}{n(i_{All\_corpus},all)+1}$ （1）

where $n(i_{C\_corpus},j)$ is the occurrence frequency of semantic primitive *i* and its similar semantic primitive in the *j* th text; n (all) is the number of texts in the corpus; and $n(i_{All\_corpus},all)$ is the occurrence frequency of semantic primitive *i* in all texts in the corpus. When calculating $n (i_{{C\_}_{corpus}},j)$ and $n (i_{{All\_}_{corpus}},all)$, we used the generated Word2Vec model. The expressions of $n (i_{{C\_}_{corpus}},j)$ and $n (i_{{All\_}_{corpus}},all)$ are as follows:

$n(i_{C\_corpus},j)=\sum\left\{termword\_num(i)|\cos(i,k)>s,k\in j,j\in C\_corpus\right\}$ (2)

$n(i_{All\_corpus},all)=\sum\left\{C\_corpus\_num(i)|\cos(i,d)>s,d\in all,all\in All\_corpus\right\}$ (3)

where *k* is a random semantic primitive in the *j* th post; *d* is a random semantic primitive in a random kind of post revealing reasons for inferiority; *s* is the experiment threshold; *C_corpus* is a random category of posts; *All_corpus* is all posts; and $\cos(\alpha,\beta)$ is the similarity of semantic primitive *i* and semantic primitive *k*. Given that each semantic primitive is a 400-dimensional vector, this study evaluated the similarity between two vectors by measuring the cosine of the angle between them in the inner product space, and the formula is as follows:

$\cos\theta=\cos(\alpha,\beta)=\frac{\sum_{r=1}^{n} (i_{r}\times k_{r})}{\sqrt{\sum_{r=1}^{n} i_{r}^{2}}\times\sqrt{\sum_{r=1}^{n} k_{r}^{2}}}$ (4)

where *i* and *k* are the n-dimensional vectors $\alpha(i_{1},i_{2},i_{3},...,i_{n})$ and $\beta(k_{1},k_{2},k_{3},...,k_{n})$, respectively;$\cos\theta\in[-1,1]$, if it is -1, it indicates that the two words are antonyms, if it is 0, it means that two words have no semantic repetition , if it is 1, it represents semantic equality between two words.

When the similarity threshold *s* value of formula (4) is 0.90 across many tests, we can obtain a relatively accurate semantic similarity result and select the words whose *SF-SAI* values are in the first 30% for semantic analysis.

## Appendix 2: Word dimension reduction visualization

The semantic primitives extracted by the *SF-SAI* algorithm were 400-dimensional vectors, which could not conveniently be used to intuitively describe the spatial distribution characteristics of the semantic primitives. Therefore, it was necessary to map the high-dimensional semantic primitive vectors to the two-dimensional plane for visual analysis. The specific steps are as follows:

First, we used the *t-SNE* algorithm to map the 400-dimensional semantic primitive vectors to two-dimensional vectors. *t-SNE* is a nonlinear dimensional simplification method based on machine learning, and its core purpose is to reduce high-dimensional data into two-dimensional space (Table 3). Notably, when there is information missing in the dimension reduction process, the *t-SNE* algorithm maintains the distance relations between high-dimensional vector points ^61,62^.

Second, when we imported the semantic primitives reduced to two-dimensional vectors into the two-dimensional visualization software, each semantic primitive was presented as a point in two-dimensional space, and the distance between two semantic primitives was proportional to the similarity of their semantics. However, it is insufficient to merely visualize the distances between semantic primitives to express the semantic range of semantic primitives, as it impedes the exploration of the semantic relations between semantic primitives. Thus, it is crucial to quantify the degrees of correlation between semantic primitives. Therefore, we used Sklearn0.19.0 in Python to conduct core density analysis on the semantic primitives and Seaborn0.8.1 drawing tool to conduct visualization.

| Table 3 Examples of reduced two-dimensional word vectors | | | | |
| --- | --- | --- | --- | --- |
| **ID** | **Semantic primitive** | **X** | **Y** | **English** |
| 1 | 傲气 | -0.859604166 | 1.984212902 | Arrogant |
| 2 | 倔强 | 0.643495166 | -1.313592229 | Obstinate |
| 3 | 天生 | -0.573996029 | -1.886607475 | Innate |
| 4 | 偏执 | 1.278016735 | -0.601750412 | Stubborn |
| 5 | 强势 | 0.515507556 | 1.197673322 | Mighty |

## Appendix 3: Inferiority feelings about personality

 (Drawing with Seaborn 0.8.1 in python^37,38^)

## Appendix 4: Inferiority feelings about social interaction

 (Drawing with Seaborn 0.8.1 in python^37,38^)
